# Exploring the risk stratification of carotid plaque hemodynamic using four-dimensional blood flow technology

**DOI:** 10.3389/fmolb.2025.1685377

**Published:** 2026-01-15

**Authors:** Si-yuan Zhang, Hong-liang Zhao, Xiao-wei Song, Jian Wu, Rui Li

**Affiliations:** 1 The First Affiliated Hospital of Sun Yat-sen University, Guangzhou, China; 2 Beijing Tsinghua Changgung Hospital, Affiliated to Tsinghua University, Beijing, China; 3 IDG-McGowan Institute of Brain Science, Tsinghua University, Beijing, China; 4 Center for Biomedical Imaging Research, Department of Biomedical Engineering, School of Medicine, Tsinghua University, Beijing, China

**Keywords:** 4D-flow magnetic resonance imaging, high-resolution magnetic resonance imaging, carotid artery plaque, hemodynamics, risk stratification

## Abstract

**Introduction:**

Carotid plaque rupture is a major cause of cerebrovascular events. This study explores the integration of hemodynamic parameters with structural biomarkers for improved risk stratification.

**Methods:**

Fifty-seven patients with moderate-to-severe carotid stenosis underwent 4D-flow magnetic resonance imaging (MRI) and high-resolution MRI. Hemodynamic parameters [wall shear stress (WSS) and velocity] were analyzed using GT-Flow software at upstream, throat, and downstream plaque regions. After comparison of characteristic values between symptomatic and asymptomatic plaques, variables with *p* < 0.1 were included in the multivariate logistic regression model to identify independent risk factors.

**Results:**

WSS was significantly higher at plaque throat (0.891 ± 0.422 Pa) and downstream (0.971 ± 0.587 Pa) versus upstream (0.649 ± 0.297 Pa; *p* < 0.001). Symptomatic plaques showed elevated 3D-WSSmean (1.041 ± 0.418 vs. 0.797 ± 0.402 Pa, *p* = 0.032), WSS up_max (1.345 ± 0.570 vs. 0.970 ± 0.383 Pa, *p* = 0.004), and stenosis velocity (31.7 ± 9.9 vs. 25.8 ± 10.3 cm/s, *p* = 0.036). The thin fibrous cap (TFC, OR = 5.34, *p* = 0.007) and normalized wall index (NWI, adjusted OR = 59.89, *p* = 0.029) independently predicted symptomatic plaques. The combined model (NWI + TFC + WSS down_max) predicted cerebral ischemic events within 6 months with an AUC of 0.809 (95% CI: 0.699–0.919).

**Conclusion:**

Integration of downstream hemodynamic profiling (WSS) with structural biomarkers (TFC and NWI) provides a robust stratification tool for cerebrovascular risk assessment. These quantitative parameters offer potential as molecular diagnostics for plaque vulnerability.

## 1 Introduction

Previous histopathological studies on atherosclerosis biopsies have revealed that histological subtyping provides limited prognostic information: overall histological categories remain similar between patients with different symptom types, and predictions of plaque occurrence, progression, and rupture based on histological subtyping are not entirely reliable (Redgrave et al., 2006). Moreover, further pathological research has demonstrated an association between histological features of plaques in symptomatic carotid artery stenosis patients (such as lipid core size or fibrous cap inflammation) and plaque outcomes (Redgrave et al., 2006; Howard et al., 2015; Fuchs et al., 2017). Particularly insightful is recent research indicating that once hemodynamic parameters are introduced, the prognostic role of plaque structural characteristics diminishes. Quantitative comparisons have shown that carotid arteries with a higher NWI have a 3.472-fold likelihood of stroke association, while elevated WSS increases the risk of stroke by 6.974-fold, highlighting the potentially greater impact of hemodynamic factors represented by WSS on plaque prognosis (Jia et al., 2017).

Currently, the consensus for distinguishing high- and low-risk carotid artery plaques is primarily based on imaging structural features (Cai et al., 2002; Gao et al., 2011; Saba et al., 2018). However, the influence of hemodynamic factors on the progression of atherosclerotic disease is increasingly emphasized and significantly correlated with prognosis. 4D-flow MRI provides distinct hemodynamic advantages over traditional modalities. Unlike ultrasound (limited by operator dependency and 2D sampling) or Computed Tomography Angiography (CTA) (providing static anatomical assessment only), this technology enables volumetric quantification of time-resolved 3D flow patterns throughout the cardiac cycle. Crucially, it permits direct calculation of multidirectional wall shear stress vectors—including axial, circumferential, and 3D resultant forces—which cannot be derived from conventional imaging. This capability for dynamic hemodynamic profiling offers unique insights into the biomechanical triggers of plaque vulnerability. Therefore, this study aims to utilize 4D flow technology and high-resolution magnetic resonance imaging (HR-MRI) to comprehensively characterize the structure (imaging biomarkers) and non-structural determinants (hemodynamic factors) of carotid artery plaques, enhancing our understanding of their involvement in stroke occurrence.

Our objective is to explore hemodynamic risk stratification of carotid artery plaques based on differential patient outcomes. Through this approach, we can further develop tailored imaging surveillance and intervention strategies for different populations to reduce the risk of stroke recurrence and other adverse outcomes.

## 2 Methods

### 2.1 Study design and participants

This study is a single-center prospective observational study, selecting patients confirmed to have carotid artery stenosis through screening with carotid ultrasound, CTA, or Digital Subtraction Angiography (DSA) as the study subjects. All participants met the following criteria: (1) aged >18 years; (2) presence of one or more atherosclerosis risk factors, including hypertension [systolic blood pressure (SBP) ≥140 mmHg, diastolic blood pressure (DBP) ≥90 mmHg, or self-reported use of any antihypertensive medication in the past 2 weeks], diabetes (fasting blood glucose ≥7 mmol/L, non-fasting blood glucose ≥11.1 mmol/L, or use of antidiabetic medication), or hyperlipidemia [total cholesterol (TC) ≥240 mg/dL, low-density lipoprotein cholesterol (LDL-C) ≥160 mg/dL, or high-density lipoprotein cholesterol (HDL-C) <40 mg/dL or lipid-lowering medication use]; and (3) asymptomatic carotid stenosis patients who developed symptoms or new ischemic stroke within 6 months. The exclusion criteria were rigorously applied, leading to the exclusion of 47 patients for the following reasons: (1) significant intracranial artery stenosis (>50%) on MRA (n = 9); (2) ischemic stroke of cardioembolic origin (n = 13); (3) non-atherosclerotic etiology, such as dissection or vasculitis (n = 8); (4) prior treatment with bypass or stenting before enrollment (n = 6); and (5) poor image quality (image quality score = 1) that precluded reliable analysis (n = 11).

Consequently, 57 patients with 81 carotid arteries were included in the final analysis (Figure 2). All patients underwent detailed clinical assessments, including comprehensive physical examinations, lifestyle evaluations, present illness history, and past medical history before enrollment. Patients underwent head MRI, High-Resolution Vessel Wall (HRVW)-MRI of the neck, and 4D flow MRI scans separately. HRVW-MRI collected imaging biomarkers, including stenosis severity, plaque length, lumen area, outer diameter area, normalized wall index (NWI) (plaque burden), eccentric index (EI), lipid-rich necrotic core (LRNC), intraplaque hemorrhage (IPH), thin fibrous cap (TFC), calcification, plaque surface irregularity, and plaque imaging subtype. 4D flow collected WSS (3D and axial), flow, and velocity at different positions of the plaque. The individual outcome was defined as positive if there was a new cerebrovascular event (clinically diagnosed TIA or homolateral brain infarction observed on DWI) within 6 months.

### 2.2 Imaging protocol

All participants underwent 4D blood flow MRI (independently developed) (Sun et al., 2023), HRVW 3D T1- and T2-weighted imaging (T1WI and T2WI), 3D MERGE, SNAP sequence imaging, and cranial MRI. The scans were performed using a Philips Ingenia CX 3.0T MR scanner, with an 8-channel phased-array carotid plaque coil dedicated for scanning. The coronal 3D MERGE sequence had a TR/TE of shortest/shortest, an Field Of View (FOV) of 200 mm × 159 mm, a slice thickness of 0.8 mm, an interslice gap of −0.4 mm, a voxel size of 0.8 mm × 0.8 mm × 0.8 mm, a flip angle of 6°, fat suppression using the spectral presaturation with inversion recovery (SPIR) technique, and a scan time of 4 min 50 s. The coronal 3D T1 VISTA sequence had a TR/TE of shortest/shortest, an FOV of 250 mm × 181 mm, a slice thickness of 0.6 mm, an interslice gap of −0.3 mm, a voxel size of 0.6 mm × 0.6 mm × 0.6 mm, an echo train length of 37, a flip angle of 90°, fat suppression using the spectral attenuated inversion recovery (SPAIR) technique, and a scan time of 1 min 48 s. The coronal 3D T2 VISTA sequence had a TR/TE of 1,600 m/shortest, an FOV of 250 mm × 160 mm, a slice thickness of 0.6 mm, an interslice gap of 0 mm, a voxel size of 0.6 mm × 0.6 mm × 0.6 mm, an echo train length of 50, a flip angle of 90°, the SPAIR technique, and a scan time of 5 min 17 s. The coronal 3D SNAP sequence had a TR/TE of shortest/shortest, an FOV of 200 mm × 159 mm, a slice thickness of 0.8 mm, an interslice gap of 0 mm, a voxel size of 0.8 mm × 0.8 mm × 0.8 mm, a flip angle of 11°, and a scan time of 2 min 36 s. The axial 3D QFLOW sequence had a TR/TE of shortest/shortest, an FOV of 160 mm × 160 mm, a slice thickness of 1.2 mm, an interslice gap of 0 mm, a voxel size of 1 mm × 1 mm × 1.2 mm, a flip angle of 20°, and a single acquisition using the phase contrast (PC) method for volumetric and time-resolved contrast; velocity encoding (VENC) was set at 90 cm/s to avoid aliasing artifacts, and the data were reconstructed into 15 frames using view sharing. The scan time was 11 min 27 s, depending on each participant’s heart rate.

### 2.3 Image analysis and post-processing

**FIGURE 1:**
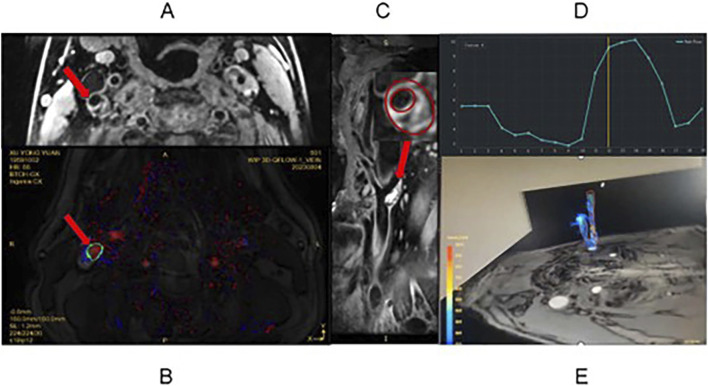
Periodic changes in wall shear stress on the carotid artery wall—schematic representation of 4D-flow characterization. **(A)** HRVW T1 image of the carotid artery, showing a ruptured fibrous cap on the plaque. **(B)** VEIN image on the 3D-Qflow sequence, displaying through-plane blood flow in the carotid artery (shown in red) in magnitude. **(C)** Sagittal reconstruction of the carotid artery from the HRVW 3D-T1 image (original image in axial view), showing the existence of IPH, calcification, and thin fibrous caps. Parameters such as the degree of lumen stenosis were measured at the axial image of the narrowest plane. **(D)** GT-Flow software integration of magnitude, AP, FH, and RL for the measurement of WSS, flow, velocity, and other hemodynamic parameters. **(E)** Pathlines of the carotid artery region of interest reconstructed from blood flow signals.

4D flow MRI data were imported into GTFlow software (version 3.2, GyroTools) for preprocessing and parameter calculation. The analysis includes the following steps. (1) Preprocessing: preprocessing steps include automatic offset correction, signal aliasing correction, flow direction correction, and full dynamic preview images to identify and exclude poor-quality images. (2) Segmentation: the target vessel is segmented parallel to the centerline and tracked along the vessel. (3) Calculation: the analysis plane is placed perpendicular to the narrowest part of the carotid artery centerline and dynamically adjusted within 20 time points to measure the velocity (maximum and average), flow (maximum and average), axial WSS (maximum and average), and circumferential WSS (maximum and average) (Figure 1). WSS is calculated according to the method described by Stalder et al. (2008). 3D WSS reflects the total WSS along the plane tangent to the local vessel surface and is decomposed into axial and circumferential components. Axial and circumferential WSSs represent the WSS along the flow direction and the vessel circumference. Original image data from MRI and 4D flow were independently measured by separate radiologists and neurologists in a blinded manner and averaged. The average post-processing time per patient using GTFlow software was approximately 30–35 min.

To assess the reproducibility of the measurements, inter-observer reliability was evaluated between the radiologist and the neurologist. Intraclass correlation coefficients (ICCs) were calculated using a two-way random-effects model for absolute agreement for continuous variables (WSS and structural parameters). For categorical variables (e.g., plaque classification), Cohen’s kappa (κ) statistic was used. The reliability analysis demonstrated excellent agreement between the two readers: the ICC for 3D WSS mean was 0.92 (95% CI: 0.87–0.95), for NWI was 0.89 (95% CI: 0.82–0.93), and for TFC thickness was 0.91 (95% CI: 0.85–0.94). The agreement for plaque classification (types I–VI) was substantial (κ = 0.78). These results indicate high consistency in image interpretation and parameter quantification.

### 2.4 Statistical analysis

All data were processed using SPSS software. Continuous variables are expressed as the mean ± standard deviation or median (interquartile range), and categorical variables are expressed as the ““frequency (percentage).” For continuous variables, independent sample t-tests or Mann–Whitney U tests were used to compare the data between two groups, and categorical variables were analyzed using χ^2^ tests or Fisher’s exact tests. All statistical analyses were performed using SPSS (version 25.0.0 IBM). Independent sample t-tests were used to compare quantitative data between groups (such as stenosis severity), and chi-square tests were used to compare categorical variables between groups. WSS and velocity values were found to follow a normal distribution based on normality tests. Chi-square tests were used to compare categorical data between groups. Logistic regression analysis was conducted separately for the two defined outcomes. Independent sample t-tests were used to compare WSS (maximum and average values) at different locations within the plaque and wall shear stress at rupture and non-rupture locations. The predictive efficacy of WSS in the plaque outcome and individual prognosis was evaluated using the ROC curve. Binary regression analysis was conducted for high-risk imaging features using high and low WSS as outcome variables. All hypothesis tests are two-sided, and a *p*-value less than 0.05 is considered statistically significant.

### 2.5 Sample size calculation

PASS-15 calculation: Taking WSS as the reference, a two-sided test is required with α = 0.05, that is, the confidence level (1 − alpha) = 0.95; according to the literature standard, the standard deviation = 0.08 (Zhang et al., 2021), and the allowable errors are taken as 0.05 and 0.02, respectively.

When δ = 0.02, N = 62; when δ = 0.05, N = 10.

This study included 57 cases with 81 carotid arteries, which had sufficient statistical power (≥98%) to address this issue.

## 3 Result

### 3.1 Demographic baseline characteristics of subjects

**FIGURE 2:**
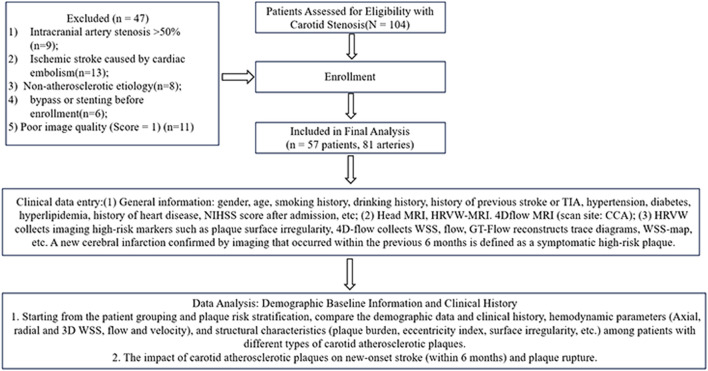
Enrollment of patients with carotid artery stenosis in this study.

The flowchart of this study is shown in Figure 2. This study included a cohort of elderly participants with an average age of 65 years. The predominant comorbidity was hypertension, observed in 68.4% of subjects, and the population was predominantly male (78.9%). A significant majority exhibited unstable plaques, with 87.7% classified as type IV–VI. The mean degree of carotid artery stenosis was 69.9% (Table 1).

**TABLE 1:** Demographic characteristics of study subjects at baseline.

Characteristic	Frequency, percentage (mean ± SD)	Characteristic	Frequency, percentage (mean ± SD)
Cohort characteristic (n = 57)		Plaque histological classification (n = 81)	
Age	65.52 ± 6.24	1–II	0, 0
Sex (male = 1)	30, 78.9	III	7, 12.3
Hypertension	26, 68.4	IV–V	24, 42.1
Diabetes	12, 31.6	VI	26, 45.6
Statin history	25, 65.8	Plaque imaging features (n = 81)	
Total cholesterol (mmol/L)	3.63 ± 0.88	IPH	27, 47.4
Triglyceride	1.23 ± 0.50	Calcification	28, 49.1
HDL-C	1.15 ± 0.28	Irregular	40, 70.2
LDL-C	2.06 ± 0.77	TRFC	34, 59.6
Smoke	24, 63.2	Lipid core area (mm^2^)	8.59 ± 12.81
Drink	22, 57.9	LRNC	37, 64.9
Plaque characteristic (n = 81)		Proportion of lipid core area	13.99% ± 0.12
Symptomatic plaque	22, 38.6	Hemodynamic parameters (n = 81)	
Stenosis	69.90% ± 0.14	3D WSS mean	0.89 ± 0.42
Length (mm)	20.47 ± 9.19	Axi WSS mean	0.42 ± 0.33
Lumen area	21.64 ± 16.50	Velocity	28.08 ± 10.45
Cross-sectional area	79.85 ± 44.41	Flow	4.49 ± 2.28
Wall area	58.20 ± 30.52	WSS up	0.65 ± 0.30
Eccentric index	0.65 ± 0.15		
NWI	0.74 ± 0.10	WSS down	0.97 ± 0.59

### 3.2 Periodic changes in carotid artery wall shear stress—characterization by 4D-flow

**FIGURE 3:**
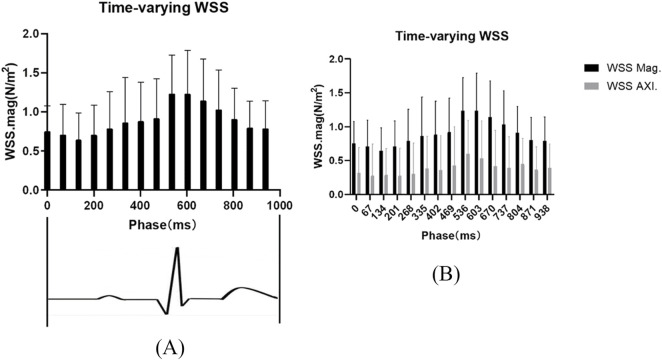
Temporal variation in carotid artery wall shear stress **(A,B)**. **(A)** Variation in the overall average value of the included carotid artery plaque measurements over time within a cardiac cycle. **(B)** Comparison of the total average values of 3D mean-WSS and Axi-WSS within a single cardiac cycle.

WSS undergoes periodic changes within a cardiac cycle, reaching its peak during the systolic phase and being at its lowest during the diastolic phase. Axial WSS changes in synchrony with 3DWSS (Figure 3).

### 3.3 Comparison of shear stress upstream, at the stenosis throat, and downstream of carotid artery plaques

**FIGURE 4:**
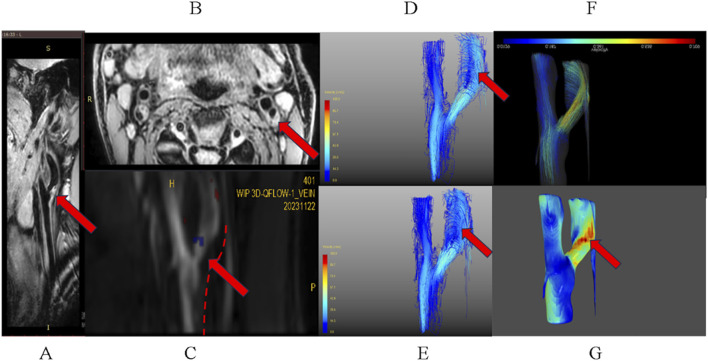
Plaque characterization with high-resolution MRI and 4D-flow hemodynamic analysis. **(A)** Coronal 3D T1 VISTA image showing a plaque at the origin of the left internal carotid artery (red arrow). **(B)** Axially reconstructed 3D-MERGE image of the same plaque. **(C)** Coronal view from GT-Flow software, showing the segmented lumen (dotted line) from 3D-Qflow data. The arrow indicates the plaque, visible as a filling defect. **(D,E)** Hemodynamic flow patterns represented by pathlines **(D)** and streamlines **(E)**, reconstructed using GT-Flow. **(F,G)** Flow patterns streamlines, **(F)** and corresponding wall shear stress map WSS-Map, **(G)** reconstructed using the R language.

WSS measurements were conducted upstream, at the stenosis throat, and downstream of the carotid artery plaques. It was found that for both the average and maximum wall shear stress, the average and maximum wall shear stress at the stenosis throat and downstream of the plaque were significantly higher than those upstream of the plaque (all *p* < 0.05), while there was no significant difference in the average and maximum WSSs between the stenosis throat and downstream of the plaque (*p* = 0.795; *p* = 0.677). Although the differences were not significant, both the average and maximum WSSs at the downstream of the plaque (3DWSSmean 0.971 ± 0.587 N/m^2^ vs. 3DWSSmax 1.639 ± 0.879 N/m^2^) were higher than those at the stenosis throat of the plaque (3DWSSmean 0.891 ± 0.422 N/m^2^ vs. 3DWSSmax 1.492 ± 0.879 N/m^2^) (Table 2; Figure 4).

**TABLE 2:** Comparison of 3D mean and maximum wall shear stress at different locations of carotid plaques.

The measured indicators	Location in plaques (I) (J)		Mean difference (I-J)	Standard error	Significance	95% confidence interval		F	Sig.
3D mean-WSS	Plaque throat	Plaque up	0.242	0.068	0.002	0.076	0.408	7.857	0.001
	(0.891 ± 0.422)	Plaque down	−0.079	0.096	0.795	−0.312	0.153		
	Plaque upstream	Plaque throat	−0.242	0.068	0.002	−0.408	0.076		
	(0.649 ± 0.297)	Plaque down	−0.322	0.087	0.001	−0.534	0.109		
	Plaque downstream	Plaque throat	0.079	0.096	0.795	−0.153	0.312		
	(0.971 ± 0.587)	Plaque up	0.321	0.087	0.001	−0.109	0.534		
3D max-WSS	Plaque throat	Plaque up	0.377	0.108	0.002	0.114	0.639	8.658	0.000
	(1.492 ± 0.650)	Plaque down	−0.146	0.144	0.677	−0.498	0.205		
	Plaque upstream	Plaque throat	−0.377	0.108	0.002	−0.640	−0.114		
	(1.115 ± 0.495)	Plaque down	−0.523	0.134	0.001	−0.849	−0.198		
	Plaque downstream	Plaque throat	0.146	0.145	0.677	−0.205	0.498		
	(1.639 ± 0.879)	Plaque up	0.523	0.134	0.001	0.198	0.849		

Plaque up represents the plaque upstream; plaque down represents the plaque downstream.

### 3.4 Comparison of inter-group characteristic values of symptomatic plaques based on the clinical definition of new ischemic events

Compared with the non-symptomatic plaque group, the symptomatic plaque group showed significant differences in imaging markers (Table 3): stenosis degree, lumen area, and NWI (plaque burden) (*p* < 0.05). However, when comparing with the imaging markers, more wall shear stress-related hemodynamic markers, such as 3D WSS mean (*p* = 0.032), 3D WSS max (*p* = 0.014), Axi WSS max (*p* = 0.079), upstream average WSS (*p* = 0.010), upstream maximum WSS (*p* = 0.004), and stenosis velocity (*p* = 0.036) showed significant differences between symptomatic and non-symptomatic high-risk plaques based on the definition of clinical ischemic events. Variables with *p* < 0.1 were included in the multivariate logistic regression model.

**TABLE 3:** Comparison of characteristic values of symptomatic plaques based on the clinical definition of new ischemic events (continuous variables).

Characteristic	Ischemia group (n = 32)	Non-ischemia group (n = 49)	Sig (bilateral)	Mean difference	Standard error difference	95% CI	
						Lower limit	Higher limit
Plaque characteristic							
Stenosis	0.756 ± 0.147	0.662 ± 0.115	**0.009**	−0.094	0.035	−0.164	−0.025
Length (mm)	20.781 ± 12.021	20.277 ± 7.052	0.842	−0.505	2.522	−5.559	4.549
Lumen area (cm^2^)	16.350 ± 14.997	24.968 ± 16.731	**0.054**	8.619	4.378	−0.155	17.392
CSA (cm^2^)	75.259 ± 46.513	82.729 ± 43.473	0.541	7.469	12.150	−16.881	31.820
Wall area (cm^2^)	58.909 ± 33.801	57.760 ± 28.775	0.891	−1.149	8.378	−17.938	15.640
Eccentric index	0.679 ± 0.130	0.632 ± 0.162	0.256	−0.047	0.041	−0.129	0.035
NWI	0.794 ± 0.098	0.706 ± 0.086	**0.001**	−0.088	0.025	−0.137	−0.038
Lipid core area (mm^2^)	10.763 ± 8.105	7.221 ± 7.929	0.314	−3.542	3.485	−10.525	3.441
Proportion of lipid core area	0.141 ± 0.120	0.139 ± 0.128	0.968	−0.001	0.034	−0.069	0.067
Hemodynamic parameter							
3D WSS min	0.580 ± 0.296	0.472 ± 0.276	0.968	−0.001	0.034	−0.069	0.067
3D WSS mean	1.041 ± 0.418	0.797 ± 0.402	**0.032**	−0.244	0.111	−0.467	−0.022
3D WSS max	1.754 ± 0.663	1.327 ± 0.593	**0.014**	−0.427	0.169	−0.765	−0.088
Axi WSS min	0.104 ± 0.395	0.018 ± 0.257	0.369	−0.086	0.095	−0.279	0.107
Axi WSS mean	0.497 ± 0.398	0.367 ± 0.266	0.144	−0.130	0.088	−0.307	0.046
Axi WSS max	1.105 ± 0.681	0.836 ± 0.456	**0.079**	−0.269	0.150	−0.570	0.032
WSS up mean	0.774 ± 0.345	0.570 ± 0.235	**0.010**	−0.204	0.077	−0.358	−0.050
WSS up max	1.345 ± 0.570	0.970 ± 0.383	**0.004**	−0.375	0.126	−0.628	−0.123
WSS down mean	1.067 ± 0.584	0.910 ± 0.589	0.329	−0.157	0.160	−0.477	0.163
WSS down max	1.813 ± 0.849	1.529 ± 0.893	0.240	−0.283	0.238	−0.761	0.194
Velocity	31.714 ± 9.909	25.803 ± 10.258	**0.036**	−5.910	2.755	−11.431	−0.388
Vel up	24.967 ± 8.816	22.013 ± 6.755	0.159	−2.954	2.070	−7.103	1.194
Vel down	32.267 ± 11.208	30.229 ± 13.156	0.550	−2.037	3.387	−8.824	4.750
Flow	4.175 ± 2.491	4.688 ± 2.157	0.414	0.513	0.623	−0.736	1.762
Flow up	5.101 ± 2.547	5.333 ± 1.853	0.693	0.232	0.583	−0.938	1.401
Flow down	3.395 ± 2.040	3.932 ± 2.112	0.348	0.537	0.567	−0.600	1.674

CSA, cross-sectional area; 3D WSS min/mean/max represent the minimum, average, and maximum wall shear stress resultant forces, respectively; Axi WSS min/mean/max represent the minimum, average, and maximum axial wall shear stresses, respectively; WSS up represents the upstream wall shear stress of the plaque; WSS down represents the downstream wall shear stress of the plaque; Velocity, Vel up, and Vel down represent the flow velocity at the stenosis site, upstream of the plaque, and downstream of the plaque, respectively; Flow, Flow up, and Flow down represent the flow volume at the stenosis site, upstream of the plaque, and downstream of the plaque, respectively. Bold values indicate statistical significance (p < 0.05).

In the categorical variable data between the symptomatic and asymptomatic plaque groups (Table 4), there were significant differences in drinking history (*p* = 0.075), calcification (*p* = 0.082), thin fibrous cap (*p* = 0.007), and plaque imaging classification (*p* = 0.016) between the groups. Among the calculated OR values, the baseline data’s TFC (OR = 5.344, 95% CI = 1.499–19) was the most significant risk factor.

**TABLE 4:** Comparison of eigenvalues between symptomatic and asymptomatic plaque groups (chi-square test).

Characteristic	Sig. (two-tailed)	OR	95% CI of OR	
			Lower limit	Higher limit
Cohort characteristic				
Sex	0.335	2.192	0.522	9.201
Hypertension	0.738	1.222	0.376	3.973
Diabetes	0.538	1.429	0.458	4.453
Smoke	0.235	2.000	0.632	6.333
Drink	**0.075**	2.824	0.895	8.906
Statin history	0.465	0.662	0.218	2.008
Plaque imaging feature				
IPH	0.160	2.167	0.731	6.419
Calcification	**0.082**	2.625	0.873	7.893
Irregular	0.738	1.222	0.376	3.973
TRFC	**0.007**	5.344	1.499	19.052
LRNC	0.682	1.266	0.409	3.917
Classification	**0.016**	-	-	-

Bold values indicate statistical significance (p < 0.1).

There was a nearly significant difference in the imaging classification of plaques (I–VI) between symptomatic plaques and asymptomatic plaques (one-way ANOVA, *p* = 0.054) (Table 5). The proportion of unstable plaques (IV–V and VI) in the high-risk plaque group was significantly higher than that of stable plaques (I–II) (*p* = 0.003 vs. *p* < 0.001). However, there was no significant difference in the proportion between hemorrhagic plaques and non-hemorrhagic plaques among unstable plaques (*p* = 0.766).

**TABLE 5:** Comparison of the imaging classification of plaques between the symptomatic plaque group and the asymptomatic plaque group.

Plaque histological classification		Mean difference (I-J)	Standard error	Significance	95% confidence interval		F	Sig.
I–II	IV–V	−0.375	0.101	**0.003**	−0.63	−0.12	3.081	0.054
	VI	−0.500	0.100	**0.000**	−0.76	−0.24		
IV–V	I–II	0.375	0.101	**0.003**	0.12	0.63		
	VI	−0.125	0.142	0.766	−0.48	0.23		
VI	I–II	0.500	0.100	**0.000**	0.24	0.76		

Bold values indicate statistical significance (p < 0.05).

### 3.5 Binary logistic regression analysis of symptomatic plaques based on the clinical definition of new ischemic events

By comparing the above inter-group indicators, parameters with *p* < 0.1 were selected [stenosis degree (*p* = 0.009), lumen area (*p* = 0.054), NWI (*p* = 0.001), 3D WSS mean (*p* = 0.032), 3D WSS max (*p* = 0.014), Axi WSS max (*p* = 0.079), WSS up mean (*p* = 0.010), WSS up max (*p* = 0.004), velocity (*p* = 0.036), calcification, plaque imaging classification, and TFC], and the imaging parameters of interest IPH and WSS down max were included in the regression analysis. The WSS parameters at the same site of the plaque were only included in the logistic regression model as the maximum value.

Binary stepwise regression showed that only the plaque imaging markers—NWI (*p* = 0.043) and TFC (*p* = 0.021)—were independent risk factors for symptomatic high-risk plaques based on clinical ischemic events (Table 6).

**TABLE 6:** Symptomatic plaque defined by clinical new ischemic events—binary logistic regression analysis.

Characteristic	B	Standard error	Significance	OR	95% CI of OR	
Plaque radiologic characteristic						
Plaque classification	1.029	0.988	0.298	2.799	0.403	19.428
Stenosis	−0.494	4.551	0.914	0.610	0.000	45.468
Lumen area	0.064	0.045	0.153	1.066	0.977	1.164
NWI	22.023	10.863	**0.043**	36.292	2.081	64.000
IPH	−1.630	1.331	0.221	0.196	0.014	2.659
Calcification	−0.528	0.927	0.569	0.590	0.096	3.627
TRFC	2.262	0.978	**0.021**	9.600	1.411	65.332
Hemodynamic index						
3DWSS mean	0.076	4.001	0.985	1.079	0.000	2743.951
3DWSS max	0.579	2.276	0.799	1.784	0.021	154.361
WSS up mean	−1.909	4.868	0.695	0.148	0.000	2064.533
WSS up max	2.073	2.642	0.433	7.951	0.045	1410.768
WSS down mean	−2.232	2.335	0.339	0.107	0.001	10.437
WSS down max	0.644	1.286	0.309	1.904	0.153	23.663
Axi WSS max	1.044	1.045	0.318	2.842	0.367	22.032
Velocity narrow	0.086	0.084	0.302	1.090	0.925	1.284

Velocity narrowing indicates the flow velocity at the smallest lumen area. The remaining hemodynamic parameters are the same as those in Table 3.

Combining the inter-group comparison and regression analysis, if the indicators of asymptomatic patients, including stenosis degree, lumen area, NWI, 3D WSS mean, 3D WSS max, Axi WSS max, WSS up mean, WSS up max, changes in the flow velocity at the stenosis site, and unstable plaques (types IV–VI), are abnormal, especially when NWI or TFC occurs, it is necessary to be vigilant about the progression of the plaque and the recurrence of ischemic events.

### 3.6 Diagnosis of multicollinearity

As shown in Table 7, after reducing the independent variables included in the model, none of the variables had a tolerance (Tol) of less than 0.1 or a variance inflation factor (VIF) greater than 10, indicating that multicollinearity did not exist. A robustness analysis was conducted by correcting for confounding factors using logistic regression.

**TABLE 7:** Diagnosis of multicollinearity in binary logistic regression variables of symptomatic plaques.

Characteristic	Unstandardized coefficient		Standardization coefficient	t	Sig.	Collinearity statistics	
	B	Standard error	Beta			Tolerance	VIF
Plaque classification	0.065	0.133	0.092	0.488	0.628	0.383	2.613
Stenosis	0.277	0.664	0.076	0.418	0.678	0.403	2.484
Wall area	0.006	0.006	0.207	1.058	0.296	0.350	2.856
NWI	2.142	1.159	0.435	1.849	0.071	0.243	4.117
IPH	−0.130	0.172	−0.134	−0.759	0.452	0.432	2.313
Calcification	−0.054	0.134	−0.055	−0.401	0.690	0.709	1.410
TFC	0.287	0.129	0.289	2.229	0.031	0.799	1.251
3DWSSmax	0.141	0.214	0.186	0.658	0.514	0.168	5.961
WSS up max	0.238	0.155	0.239	1.530	0.133	0.551	1.816
WSS down max	−0.099	0.105	−0.176	−0.942	0.352	0.383	2.611
Axi WSS max	0.042	0.156	0.048	0.268	0.790	0.420	2.382
Velocity	0.004	0.010	0.077	0.365	0.717	0.304	3.285

### 3.7 Robustness analysis—adjustment for confounding factors

After correcting for confounding factors (degree of stenosis, IPH, calcification, and imaging classification of plaques), the binary logistic regression analysis once again confirmed that the imaging markers of plaques, namely, NWI (*p* = 0.029) and TFC (*p* = 0.020), were independent risk factors for symptomatic high-risk plaques according to the definition of clinical ischemic events (Table 8).

**TABLE 8:** Binary logistic regression analysis for correcting the symptoms of plaques affected by confounding factors.

Characteristic	B	Standard error	Sig.		OR	95% CI of the OR value
Plaque radiologic characteristic						
Plaque classification	−20.358	12424.522	0.999	0.000	0.000	-
Stenosis	−1.393	4.632	0.764	0.248	0.000	217.168
Lumen area	0.072	0.046	0.119	1.075	0.982	1.178
NWI	24.816	11.593	**0.032**	59.890	8.119	441.000
IPH	1.195	1.323	0.366	3.304	0.247	44.184
Calcification	0.798	0.957	0.405	2.221	0.340	14.501
TFC	−2.360	1.013	**0.020**	0.094	0.013	0.687
Hemodynamic index						
3D WSS max	0.819	1.383	0.554	2.269	0.151	34.122
WSS up max	1.288	1.145	0.261	3.627	0.384	34.233
WSS down max	−0.639	0.628	0.309	0.528	0.154	1.807
Axi WSS max	1.029	1.032	0.319	2.797	0.370	21.141
Velocity	0.031	0.069	0.656	1.031	0.901	1.181

### 3.8 Predictive efficacy of carotid plaque wall shear stress and independent risk factors for new-onset stroke

**FIGURE 5:**
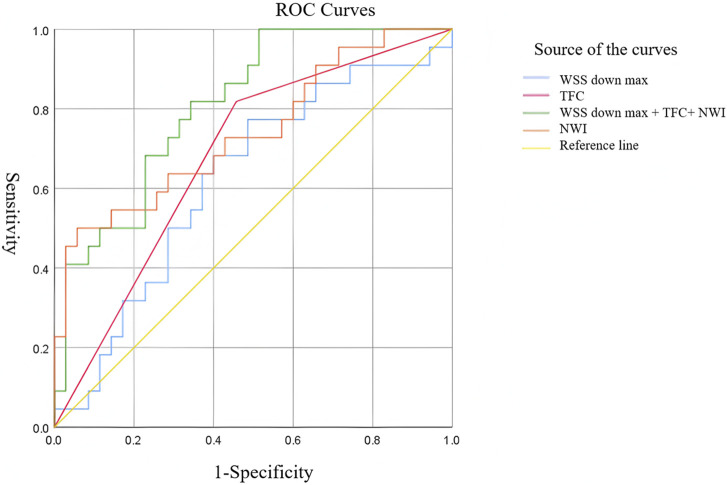
ROC curves illustrating the predictive efficacy of imaging high-risk features and hemodynamic parameters for new-onset stroke. The graph compares the diagnostic performance of four models: (1) WSS down max (blue), representing wall shear stress-derived high-risk features; (2) TFC (red), indicating hemodynamic parameters; (3) a combined model integrating WSS down max, TFC, and NWI (green); and (4) NWI alone (orange). Among individual markers, the combined model (WSS down max + TFC + NWI) exhibits the highest area under the curve (AUC), suggesting superior predictive efficacy for new-onset stroke. These findings highlight the enhanced prognostic value of multimodal integration of imaging and hemodynamic biomarkers over single-parameter assessments.

In the previous study, we found that the WSS down max was an independent risk factor for plaque rupture. Therefore, we incorporated this hemodynamic factor into the prediction model. The predictive ability of the downstream max value of WSS for new-onset stroke was AUC = 0.622, with a cutoff value of 1.302. NWI (AUC = 0.740) and TFC (AUC = 0.681) also showed good predictive efficacy. The combined index (NWI + TFC + downstream max of WSS) has the highest predictive ability (AUC = 0.809), significantly higher than downstream max of WSS and TFC (*p* < 0.05), suggesting that the combined screening of histology and hemodynamics is more capable of understanding the prognosis of individuals with carotid artery stenosis (Figure 5, Table 9).

**TABLE 9:** Predictive efficacy of WSS and independent risk factors for new-onset stroke.

Test variable	AUC	*p*	Standard error	Asymptotic sig.	Asymptotic 95% CI		Youden index max	Cutoff value
					Lower limit	Upper limit		
WSS down max	0.622	**0.035**	0.077	0.123	0.472	0.772	0.287	1.302
NWI	0.740	0.213	0.070	0.002	0.604	0.877	0.443	0.823
TFC	0.681	**0.020**	0.072	0.023	0.539	0.822	0.361	0.5
Joint indicator	0.809	-	0.056	0	0.699	0.919	0.486	0.225

## 4 Discussion

This study conducted detailed measurements of WSS upstream, at the throat region (the area with the smallest lumen area), and the downstream of the carotid artery plaques and discovered some interesting phenomena. The results showed that both the average WSS and the maximum WSS at the laryngeal region of the plaque and downstream of the plaque were significantly higher than those upstream of the plaque (*p* < 0.05). Notably, although not statistically significant, WSS at the downstream was numerically higher than that at the throat.

These findings differ from the research results on coronary artery plaques (Fukuyama et al., 2023; Kolodgie et al., 2004). In the coronary arteries, the downstream of the plaque has a lower WSS than the upstream due to blood flow disturbance and energy loss (Fukuyama et al., 2023). This difference between carotid artery plaques and coronary artery plaques may be due to the unique anatomical structure of the carotid artery. The corner at the bifurcation of the carotid artery may cause vortices to form downstream of the plaque, which could be one of the reasons for the higher WSS downstream of the plaque in this study. This continuous spiral flow pattern has been reported by Strecker et al. (2021), and it has been shown to be beneficial in patients with ICA stenosis ≤10% as this “zigzag” of the carotid artery can help protect the ICA from atherosclerosis (Strecker et al., 2021). Dilba characterized the regional distribution of WSS based on the baseline MRI contour and flow on symptomatic carotid arteries and also found that high WSS was concentrated in the downstream region of the plaque, which was consistent with the speculated vortex-shaped region.

To verify this hypothesis, this study conducted further analysis by reconstructing streamlines and pathlines (Figure 4). The analysis results supported our assumption that the special geometric shape at the carotid artery bifurcation did indeed form vortices inside the lumen downstream of the plaque (as indicated by the red arrows), which might be the main reason for the higher WSS downstream of the plaque. In addition to the above explanation, other factors may also affect the WSS distribution upstream and downstream of the carotid artery plaque. For example, the morphology and composition of the carotid artery plaque, the degree of vascular remodeling, and local hemodynamic characteristics could all affect the distribution of WSS (14). The significance of this result lies in the fact that it reveals the uniqueness of the WSS distribution upstream and downstream of the carotid artery plaque compared to that of coronary artery plaques, which is of great significance for understanding the progression of carotid atherosclerosis and clinical management. The downstream area of the carotid artery plaque may have higher WSS due to the helical blood flow pattern, which plays a protective role in mild atherosclerosis and makes it more prone to plaque rupture in moderate to severe carotid artery stenosis. The higher wall shear stress downstream of the plaque is associated with excessive expression of MMP-9 (Mu et al., 2022). This mechanotransduction pathway indicates that quantification of wall shear stress can serve as a non-invasive alternative indicator for molecular fragility biomarkers. Our research results indicate that the degree of stenosis, lumen area, NWI, TFC, 3D WSS mean/max, Axi WSS max, WSS up mean, WSS up max, changes in flow velocity at the stenosis site, and the occurrence of unstable plaques (types IV–VI) are all risk factors for symptomatic plaques that require vigilance. In particular, NWI and TFC have been confirmed as independent risk factors through regression analysis. Their identification as independent predictors aligns with their well-established roles in plaque instability and thromboembolism. These risk factors may affect the stability of the plaque and subsequently influence the occurrence of ischemic events.

The independent predictive values of NWI and TFC can be explained by their central roles in plaque vulnerability. A high NWI signifies substantial plaque burden, which is often associated with inflammatory activity and a larger lipid core, increasing susceptibility to rupture under hemodynamic stress (Lal et al., 2011; Tang et al., 2008; Cao et al., 2020). Concurrently, TFC represents a critical structural failure, where a thin or ruptured fibrous cap directly facilitates thrombosis and the release of emboli, leading to cerebral ischemia (Cao et al., 2020; Redgrave et al., 2006). Although WSS was not an independent factor in the final model, hemodynamic forces may indirectly contribute by influencing the morphological and compositional development of these high-risk features (Zhang et al., 2023).

The predictive power of the downstream maximum of WSS (WSS down max) for new-onset stroke showed moderate strength (AUC = 0.622), with a cutoff value of 1.302. In addition, plaque morphological indicators such as TFC and NWI also showed good predictive efficacy. The combined model (NWI + TFC + WSS down_max) achieved significantly enhanced predictive ability (AUC = 0.809), indicating that combined screening of histology and hemodynamics can more comprehensively assess the prognosis of patients with carotid artery stenosis. Our findings, enabled by 4D-flow MRI, show that WSS-based hemodynamic assessment predicts the stroke risk as effectively as structural biomarkers. This highlights its potential as a non-invasive tool for evaluating plaque vulnerability based on direct biomechanical profiling. It is recommended that in type IV–VI plaques with large plaque load, thin fibrous caps, and calcification as observed on carotid magnetic resonance imaging, mechanical indicators related to WSS be measured as a routine screening method for stroke risk. The combined model (NWI + TFC + WSS down max) achieved a clinical-grade predictive value (AUC = 0.809; cutoff = 0.225), suggesting its utility for identifying high-risk plaques requiring intensive intervention. However, implementation barriers remain: 4D-flow acquisition time (11 min 27 s) currently limits emergency applications, and standardized WSS quantification protocols are still evolving (Sun et al., 2023). Future integration of accelerated 4D-flow sequences with deep learning reconstruction could enable point-of-care hemodynamic risk stratification (Peper et al., 2020). This hemodynamic-structural paradigm shift enables the precision identification of vulnerable plaques through three synergistic biomarkers: (1) NWI quantifying plaque burden, (2) TFC indicating cap integrity, and (3) downstream WSS mapping biomechanical stress (Figure 4G). Validation in multicenter cohorts established standardized risk thresholds for clinical deployment.

This study has limitations inherent to its cross-sectional design, which precludes the establishment of causal relationships between plaque characteristics and outcomes. Furthermore, our analysis focused on the culprit plaque and did not account for the potential influence of total plaque burden or collateral circulation status on overall stroke risk. Studies have shown a significant association between the total number of plaques and the recurrence of acute stroke events (Park et al., 2019). However, the role of the number of plaques in plaque rupture has been proven to be limited (Dudanov et al., 2003). Future longitudinal studies tracking the evolution of hemodynamic and structural parameters are warranted.

## 5 Conclusions

Our findings establish 4D-flow hemodynamic profiling as a transformative diagnostic tool for cerebrovascular risk stratification. The integration of downstream wall shear stress quantification with structural biomarkers (TFC and NWI) creates a novel paradigm where biomechanical forces directly inform molecular vulnerability assessment. This synergistic approach enables the precision identification of high-risk plaques warranting targeted interventions, advancing the frontier of molecular diagnostics in cerebrovascular disorders.

## Data Availability

The raw data supporting the conclusions of this article will be made available by the authors, without undue reservation.
