# Supplementary material for: Exploring the risk stratification of carotid plaque hemodynamic using four-dimensional blood flow technology
Source: Front Mol Biosci. 2026 Jan 15;12:1685377. doi: 10.3389/fmolb.2025.1685377 (PMC12852037; doi:10.3389/fmolb.2025.1685377)
Supplement: Supplementary file 1 [file DataSheet1.pdf]

Table S1. Comparison of Symptomatic Plaques (TIA-only) vs. Asymptomatic Plaques

Characteristics	TIA-only Group (n=10)	Asymptomatic Group (n=49)	p-value
**Plaque Characteristics**			
Stenosis degree	0.74±0.15	0.66±0.12	**0.028**
Lumen area (cm²)	17.85±15.21	24.97±16.73	0.098
NWI	0.78±0.10	0.71±0.09	**0.009**
**Hemodynamic Parameters**			
3D WSS mean (Pa)	1.02±0.43	0.80±0.40	**0.047**
WSS up max (Pa)	1.31±0.59	0.97±0.38	**0.012**
Stenosis Velocity (cm/s)	30.9±10.1	25.8±10.3	0.078
**Imaging Features**			
Thin Fibrous Cap (TFC), n(%)	6 (60.0%)	15 (30.6%)	**0.015**
Calcification, n(%)	5 (50.0%)	19 (38.8%)	0.105

Note: Data are presented as mean ± standard deviation or frequency (percentage).
NWI: Normalized Wall Index; WSS: Wall Shear Stress.
The p-values for continuous variables are from independent samples t-test; for categorical variables, from Chi-square test.

Table S2. Comparison of Symptomatic Plaques (Stroke-only) vs. Asymptomatic Plaques

Characteristics	Stroke-only Group (n=22)	Asymptomatic Group (n=49)	p-value
**Plaque Characteristics**			
Stenosis degree	0.77±0.14	0.66±0.12	**0.002**
Lumen area (cm²)	15.42±14.88	24.97±16.73	**0.018**
NWI	0.80±0.09	0.71±0.09	**<0.001**
**Hemodynamic Parameters**			
3D WSS mean (Pa)	1.05±0.41	0.80±0.40	**0.016**
WSS up max (Pa)	1.36±0.56	0.97±0.38	**0.002**
Stenosis Velocity (cm/s)	32.2±9.8	25.8±10.3	**0.019**
**Imaging Features**			
Thin Fibrous Cap (TFC), n(%)	18 (81.8%)	15 (30.6%)	**<0.001**
Calcification, n(%)	10 (45.5%)	19 (38.8%)	0.154

Note: Data are presented as mean ± standard deviation or frequency (percentage).
NWI: Normalized Wall Index; WSS: Wall Shear Stress.
The p-values for continuous variables are from independent samples t-test; for categorical variables, from Chi-square test.

Table S3. Inter-observer Reliability for Hemodynamic and Structural Parameters

Parameter	Assessor 1 (Mean ± SD)	Assessor 2 (Mean ± SD)	ICC (95% Confidence Interval)	Reliability Level
**Hemodynamic Parameters**				
3D WSS mean (Pa)	0.89 ± 0.42	0.86 ± 0.45	0.92 (0.87 – 0.95)	Excellent
3D WSS max (Pa)	1.49 ± 0.65	1.52 ± 0.70	0.90 (0.84 – 0.94)	Excellent
WSS up max (Pa)	1.12 ± 0.50	1.08 ± 0.53	0.88 (0.81 – 0.93)	Excellent
WSS down max (Pa)	1.64 ± 0.88	1.59 ± 0.91	0.91 (0.85 – 0.94)	Excellent
**Structural Parameters**				
NWI	0.74 ± 0.10	0.73 ± 0.11	0.89 (0.82 – 0.93)	Excellent
TFC Thickness (mm)	0.45 ± 0.15	0.47 ± 0.16	0.91 (0.85 – 0.94)	Excellent
Stenosis Degree (%)	69.9 ± 14.0	68.5 ± 14.8	0.93 (0.89 – 0.96)	Excellent
**Categorical Parameter**			**Cohen's κ (95% CI)**	
Plaque Classification (I-	-	-	0.78 (0.65 –	Substantial

Parameter	Assessor 1 (Mean ± SD)	Assessor 2 (Mean ± SD)	ICC (95% Confidence Interval)	Reliability Level
VI)			0.91)	

Note: ICC, Intraclass Correlation Coefficient. ICC and κ values were interpreted as follows: <0.50, poor; 0.50–0.75, moderate; 0.75–0.90, good; and >0.90, excellent reliability. SD, Standard Deviation.
